# A high-speed electro-optic triple-microring resonator modulator

**DOI:** 10.1038/s41598-017-04851-x

**Published:** 2017-07-05

**Authors:** Jianxun Hong, Feng Qiu, Xiaoyang Cheng, Andrew M. Spring, Shiyoshi Yokoyama

**Affiliations:** 1 Institute for Materials Chemistry and Engineering, Kyushu University, 6-1 Kasuga-koen, Kasuga-city, Fukuoka 816-8580 Japan; 2 School of Information Engineering, Wuhan University of Technology, Wuhan, 430070 China

## Abstract

The coupling intensity modulator based on a triple-microring structure was proposed and numerically investigated for a high speed and a low bit error ratio (BER) operation. The modulator consists of a dual-microring optical cavity and a gate-microring energy feedback path. The optical cavity ensures a high energy storing efficiency, and the feedback path enables modulation with little intracavity energy decay. The bandwidth of 103 GHz and modulation depth of 6.2 dB at 2.0 Vpp were theoretically verified by the analysis of the sinusoidal modulation performance. Pulse modulation resulted in a data rate of 160 Gbps, an extinction ratio of 16.84 dB, and a BER of 1 × 10^−8^. The proposed modulator is applicable for compact, high-speed, and low-energy photonic integration.

## Introduction

The microring modulators are promising candidates for photonic integrated circuits due to their straightforward fabrication, compact footprint, cost effectiveness, high-sensitivity, and low driving voltage^1–6^. The principle operation of the classical microring modulator is based on a wavelength shift of the resonance due to the minute intracavity refractive index change^7^. Generally, the high quality-factor (*Q*) microring is sensitive to the intracavity refractive index, so that the device exploits a low power and high-sensitivity tuning operation^8–10^. Nevertheless, very high-*Q* microring processes a long optical cavity photon lifetime, so that the modulation speed is intrinsically limited by the optical cavity photon lifetime. Consequently, there is a crucial tradeoff between switching energy and modulation bandwidth as an unavoidable issue toward the ultra-high speed analog communications and data processing^11^.

In order to break the photon lifetime limitation, coupling intensity modulations have been proposed in the past years^12–15^. It has been theoretically predicted that the microring retains constant optical power in the cavity, while the cavity releases the light by tuning the coupling coefficient between the resonator and waveguide^13^. Since the modulator is tuned by the coupling coefficient instead of the intracavity index, the modulation frequency can be improved beyond the cavity photon lifetime limitation. There have been several proposed types of coupling intensity modulators. Mach-Zehnder interference based microring resonators (MZIMR) have demonstrated unlimited bandwidth modulation in theory^14, 16–18^. The phase-ring-enhanced MZIMR is another approach to improve the modulation frequency by using the additional phase ring adjacent to the arm of the MZI^1, 19^. These devices exhibited a high extinction and ultra-high speed response to the driving signal. This method has been further investigated for optimization^20^. Potentially, both the modulation speed and signal extinction can be further increased by using an optimized energy feedback mechanism with a greater storing effective energy and constant optical power.

In this study, we have designed a triple-ring resonator for coupling intensity modulation, and have demonstrated high-speed transmission with a large signal extinction ratio. This structure consists of an optical cavity with high energy storage efficiency and a controllable energy feedback path. The optical cavity ensures a large output intensity. The feedback path ensures a high speed modulation by keeping stable the optical cavity power. By utilizing the numerical calculation, the modulator exhibits a transmission as high as 160 Gbps, a modulation depth of 6.2 dB, an extinction ratio of 16.84 dB, and a BER of 1 × 10^−8^.

## Results and Discussion

### Principle and optical spectrum

**Figure 1:**
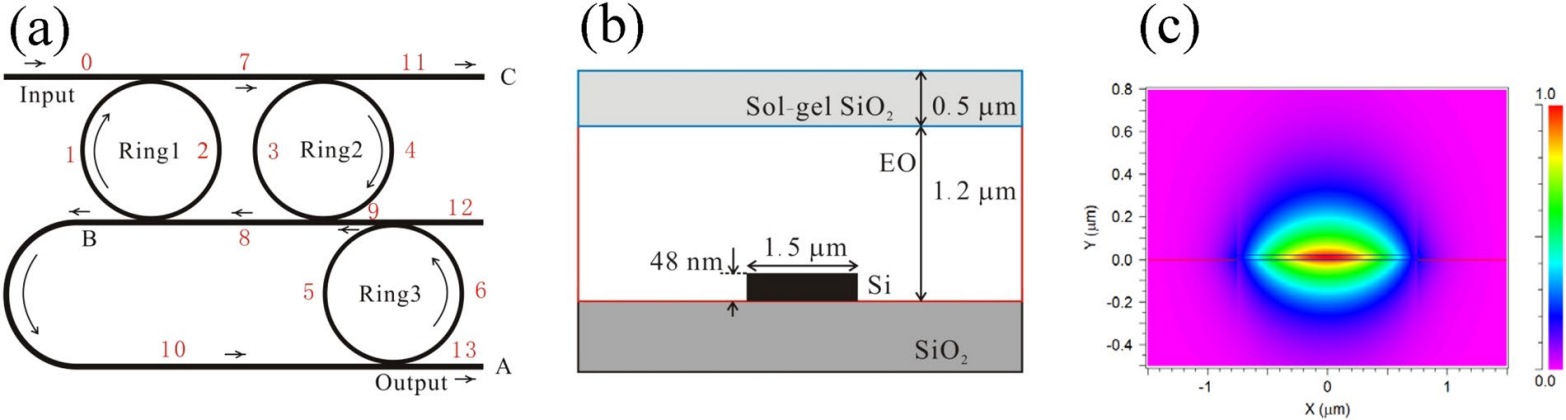
Schematics of the triple-ring resonator modulator. (**a**) Structure of the modulator. (**b**) Cross-section of the waveguide. (**c**) Fundamental TE mode distribution. The lengths of the waveguides are *L*_0_ = *L*_11_ = *L*_12_ = *L*_13_ = 40 µm, *L*_1_ = *L*_2_ = *L*_3_ = *L*_4_ = 240 µm, *L*_5_ = *L*_6_ = 120 µm, *L*_7_ = *L*_8_ = 240 µm, *L*_9_ = 80 µm, and *L*_10_ = 400 µm. The *L*_1_, *L*_2_, *L*_3_ and *L*_4_ are chosen to set the resonance close to the wavelength of 1550 nm. The modal overlap integral factor Γ is 0.525.

The microring modulator is schematically shown in Fig. 1(a). It consists of three rings 1, 2, and 3. Rings 1 and 2 have the same geometry, and work as a dual-microring cavity for optical energy storing^12^. Ring 3 is a component of the energy feedback path, and is used to develop the light from port A. When ring 3 is tuned to be on resonance, the traveling light in port B is fed back into the cavity. In this case, the resonance of ring 3 is in the same as that of rings 1 and 2. When ring 3 is tuned to be off resonance by changing the refractive index, the cavity releases the light from the feedback path to port A. Even while in such an off state, rings 1 and 2 retain sufficient photon energy in the optical cavity, which is key for coupling intensity modulation.

In order to operate the refractive index tunable microring resonator, we used a hybrid silicon and electro-optic (EO) polymer microring resonator^21^. We have previously shown that the waveguide modulator provides a large EO effect in device. The measured EO coefficient (*r*_33_) was higher than 100 pm/V at a wavelength of 1550 nm. Due to the relatively high refractive index of the hybrid waveguide, 100 μm-diameter ring resonator with a high-*Q* was realized for the application of the EO ring resonator modulator^22^. Figure 1(b) shows the cross-section of the hybrid waveguide. We used ultra-thin silicon with a thickness of 48 nm and a width of 1.5 μm as the core. This was then, covered by a 1.2 μm-thick EO polymer layer. Figure 1(c) shows the calculated TE mode distribution across the waveguide. In such geometry, the waveguide enables the light propagation at the boundary of the silicon and EO polymer layers, thus the waveguide exhibits a suitable modal overlap for the EO modulation. The distribution of the electric field is continuous in the vertical direction, while horizontal discontinuities at the boundaries of the silicon exist due to the large refractive index difference between silicon and EO polymer. Based on these experimental and theoretical properties, we utilized the step-segment dynamic method for the calculation of the coupling intensity modulation^23, 24^.

**Figure 2:**
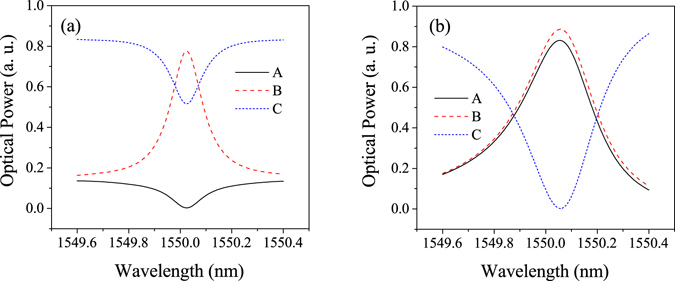
Optical spectra of the micro-ring resonator. (**a**) On resonance in ring 3 and (**b**) off resonance by adding a refractive index tuning of Δ*n* = 4 × 10^−4^.

The self-coupling coefficient (*t*_*m*_) and cross-coupling coefficient (*κ*_*m*_) at coupling points of *m* ^th^ microring are denoted by |*t*_*m*_|^2^ + |*κ*_*m*_|^2^ = 1 for a lossless coupling. For calculation of the optical transmission spectra, we chose *κ*_1_ = *κ*_2_ = *κ*_3_ = −0.4i, and transmission loss of *α* = 4 dB/cm. The effective refractive index *n*_eff_ of the waveguide is 1.643. Ring 3 is tuned owing to the EO property of the hybrid waveguide. According to the expected change in the refractive index (Δ*n* = 4 × 10^−4^) in ring 3, the spectral change of the output lights from ports A, B, and C are obtained as shown in Fig. 2. In port A, clear “ON” and “OFF” states can be seen at the center wavelength of 1550.02 nm. In the coupling intensity modulator, the dual-microring cavity stores optical energy. Therefore, the spectra of port B show relatively high-*Q* properties having 8,333 and 4,403 at the “ON” and “OFF” states, respectively. Furthermore, the modulator overcomes the cavity photon lifetime limitation.

### Sinusoidal modulation and frequency response

The high frequency modulation was analyzed by using the step-segment dynamic method. In the EO microring modulator, the relationship between the refractive index change and applied field is expressed as ${\rm{\Delta }}n=1/2{{n}_{{\rm{eff}}}}^{3}{r}_{33}{\rm{\Gamma }}E$, where *r*_33_ is the in-device EO coefficient, Γ is the modal overlap integral factor, *E* is the applied electric field^25^. For simplicity, we choose *r*_33_ = 150 pm/V to set *r*_33_Γ = 80 pm/V. We assume the electrodes in a coplanar-strip geometry having a gap of 4 µm. The velocity mismatch between the optical wave and the driving microwave can be expressed in terms of index difference. We set the index difference to be 0.2 as a compromise by taking into account the low dielectric constant of EO polymer.

**Figure 3:**
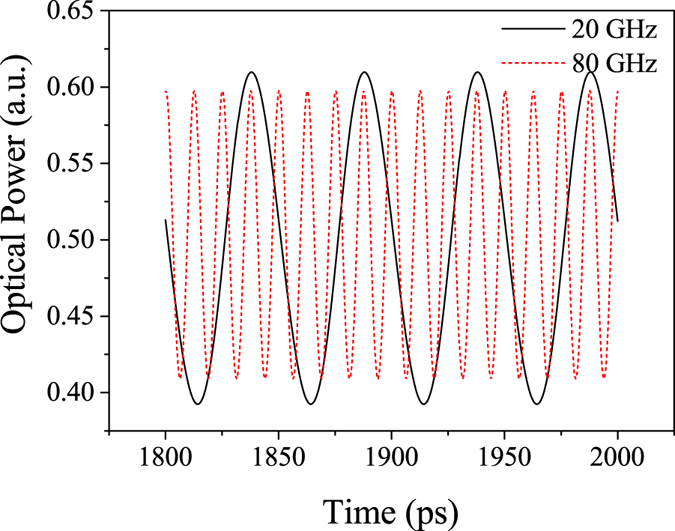
Modulated output signals of the resonator. The modulation frequencies are 20 GHz and 80 GHz. Here, the peak-to-peak voltage of the driving voltage is 2.0 V. The modulator is biased to set the output signals center at 0.5 along the longitudinal coordinate.

**Figure 4:**
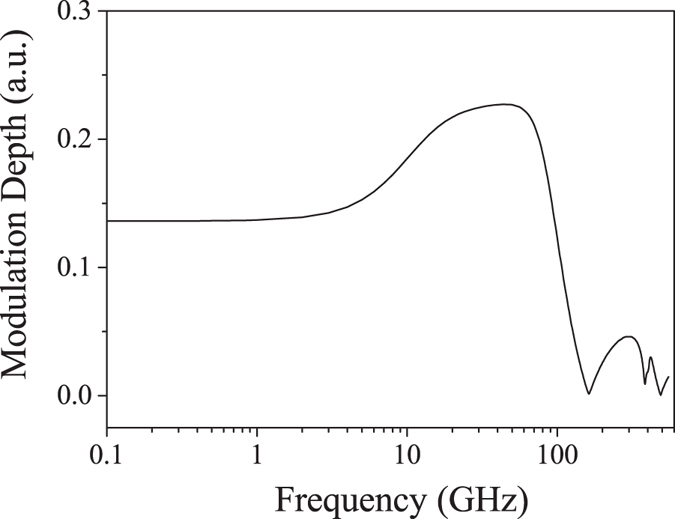
Frequency response of the resonator modulator.

A sinusoidal electric signal with a peak-to-peak voltage of 2.0 V is applied for the modulation. The modulated output signals with frequencies of 20 GHz and 80 GHz are shown in Fig. 3. A bias voltage is also applied to set the modulator at the half output point for linear electric and optic operation. The clear output waveforms can be observed at both frequencies without any distortion. The amplitude of the light intensity at the frequency of 80 GHz is slightly smaller than that at 20 GHz. In Fig. 4, the continuous frequency signals from 0.1 to 500 GHz are applied in order to determine the bandwidth property of the modulator. The maximum modulation depth is found to be 0.23 or 6.4 dB. Such modulation depth is larger than that reported for MZIMR and comparable to the phase ring enhanced MZIMR^20^. In Fig. 4, there is a plateau region between 10 GHz and 80 GHz, which offers a large bandwidth window for the high-speed modulator application. The modulation depth decreases at higher frequencies, and the modulator exhibits a 3 dB bandwidth of 103 GHz. Though MZIMR theoretically predicted an unlimited bandwidth property^14–18^, the frequency limitation in our modulator presumably results from the index modulation in ring 3. It leads to the roll-off in the modulation depth at very high modulation frequencies because the sidebands diminish with increasing modulation frequency^15, 20^. When the frequency increases close to an integer multiple of the free spectra rang (FSR), the modulation causes a significant decrease to the circulating field in the cavity^16^. In Fig. 4, the oscillations and resonance points are observed over 160 GHz. Such limitation is similar to that exists in the phase ring enhanced MZIMR^1, 20^. Note that, taking into account the roll-off effect mentioned above, the frequency at a resonance point is not exactly an integer multiple of the FSR.

### Pulse modulation properties

For the analogue sinusoidal modulation, the modulator is properly biased at the half output point as mentioned above. Therefore, the gate ring is always in ‘open’ status during modulation. In such an open state, the modulation speed is limited due to the decrease of stored energy and *Q*-factor of the cavity. On the other hand, the pulse modulation is the crucial technique, which enables the high-speed digital transmission by simple ‘0’ and ‘1’ signals. Since the bias-voltage control is unnecessary for this modulation, more energy can be stored in the cavity. In order to characterize the response to the digital signals, we firstly applied non-return-to-zero (NRZ) pulse train with alternate ‘0’ and ‘1’ to the microring modulator and calculated the output waveform. Then, we used pseudo random NRZ sequence to investigate the eye diagram and BER.

**Figure 5:**
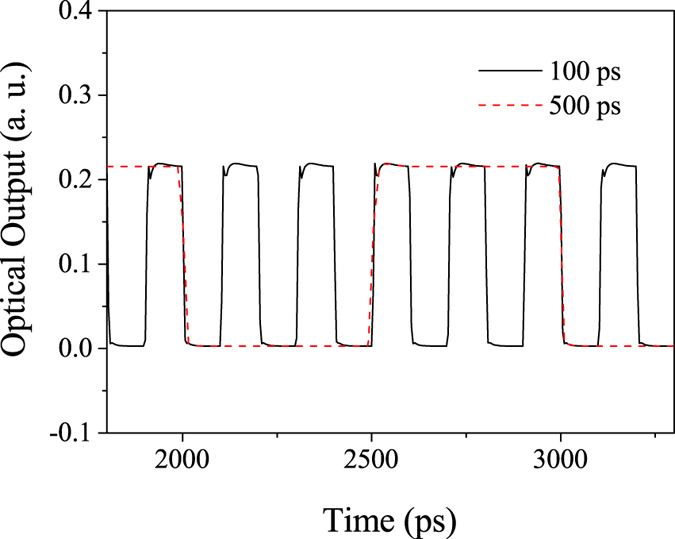
NRZ pulse train modulation output with pulse intervals of 100 ps and 500 ps. The NRZ pulse train consists of alternate ‘0’ and ‘1’ codes with a voltage of 2.0 Vpp.

Figure 5 shows the optical outputs from the modulator by applying the pulse signal with intervals of 100 ps and 500 ps, which correspond to repetition rates of 10 Gbps and 2 Gbps, respectively. The peak-to-peak voltage of the NRZ pulse train is 2.0 V. It can be seen that the amplitudes of the output lights for both signal rates are almost same. The extinction ratio is 20 dB, which is superior to the reported value in previous coupling intensity modulated MZIMR^11, 20^. In both pulse responses, small oscillations are seen at the rising edge of the signals. Such oscillation can be explained by the microring memory effect^16, 20^. In this design, the index of ring 3 is tuned instead of *κ*_3_. The circulating field in ring 3 suffers continuous index modulation and time delay. Therefore, the ring cannot response to the fast change of the pulse signal at the rising edge, and causes a short oscillation before stabilization. Such an unexpected distortion is conspicuous in the pulse waveform at the higher frequency. Though overserved noise level is small enough to be filtered out by the signal processing technique, using the smaller ring is another solution to cancel such oscillations.

**Figure 6:**
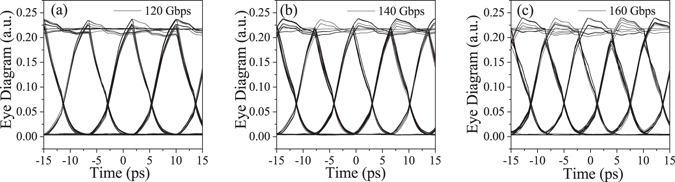
Eye diagrams of the 2^23^-1 PRBS modulation output. The modulation rates are (**a**) 120 Gbps, (**b**) 140 Gbps and (**c**) 160 Gbps, respectively.

Figure 6 show the eye diagrams of the output signal at the pulse modulation rates of 120, 140 and 160 Gbps. The pulse train consists of a 2^23^-1 pseudo random bit sequence (PRBS) signal with a peak-to-peak voltage of 2.0 V. The eye diagrams are clearly opened at every modulation rate. The modulator performed a very low BER response with clear rising and falling edges, fine code alignment and a small jitter. The BERs at these rates are calculated to be smaller than 1 × 10^−8^. On the other hand, the eye patterns became unclear with increasing BER when the modulation rate increased over 200 Gbps.

In the eye diagram analysis, the signal-to-noise ratio (*SNR*) can be obtained from ref. 26

1 $$SNR=\frac{{\mu }_{2}-{\mu }_{1}}{{\sigma }_{2}+{\sigma }_{1}}$$

where *μ*_1_ and *μ*_2_ are the eye amplitudes at the bottom and top edges, respectively. *σ*_1_ and *σ*_2_ are noises of *μ*_1_ and *μ*_2_, respectively. In Fig. 6(a), the *SNR* is estimated to be 33.33 for 120 Gbps, and the extinction ratio is 16.84 from *ER* = 10 log(*μ*_2_/*μ*_1_). According to the classical Shannon formula defined by *C* = *W* log_2_(1 + *SNR*), the modulation capacity (*C*) of the proposed microring modulator is 525 Gbps using the bandwidth (*W*) of 103 GHz. Though the calculation is theoretically based on the ideal condition, the obtained result supports the potentially available ultra-high speed response of the triple-microring resonator.

## Conclusions

We investigated a triple-microring resonator modulator based on coupling intensity modulation. The device strategy is to maintain the intracavity energy by using the dual-microring optical cavity, while the modulation is tuned by adopting a gate-microring energy feedback path. The modulation is realized by switching the feedback gate-microring between on-resonance and off-resonance states. The proposed triple-microring resonator performs a wide 3 dB bandwidth of 103 GHz and a high modulation depth of 6.4 dB at 2.0 Vpp. For digital modulation, the device exhibits a modulation speed beyond 160 Gbps with a BER of 1 × 10^−8^ and an extinction ratio of 16.84 dB. The proposed device is potentially applicable to ultra-high speed, low-energy, and small-footprint modulators.

## Methods

We use the step-segment dynamic method to analyze the modulation properties. Branches of the waveguide were divided into small segments. Fields on branches were calculated by the wave propagation principle while fields at coupling nodes were calculated by the coupled mode theory step by step^23, 24^. Numerical calculations were performed by C-language programing.
